# Cross-Sectional Investigation of HEMS Activities in Europe: A Feasibility Study

**DOI:** 10.1155/2014/201570

**Published:** 2014-11-30

**Authors:** Stefano Di Bartolomeo, Paolo Gava, Anatolij Truhlář, Mårten Sandberg, The Euphorea Group

**Affiliations:** ^1^Department of Research and Development, Norwegian Air Ambulance Foundation, Holterveien 24, P.O. Box 94, 1441 Drøbak, Norway; ^2^Azienda Ospedaliero Universitaria di Udine, Piazza SM Misericordia 15, 33100 Udine, Italy; ^3^Department of Anesthesiology and Intensive Care, Charles University in Prague, Faculty of Medicine in Hradec Kralove, University Hospital Hradec Kralove, 500 05 Hradec Kralove, Czech Republic; ^4^Air Ambulance Department, Division of Emergencies and Critical Care, Oslo University Hospital, Sykehusveien 19, 1474 Nordbyhagen, Norway

## Abstract

*Objectives.* To gather information on helicopter emergency medical services (HEMSs) activities across Europe.* Methods. *Cross-sectional data-collection on daily (15 November 2013) activities of a sample of European HEMSs. A web-based questionnaire with both open and closed questions was used, developed by experts of the European Prehospital Research Alliance (EUPHOREA).* Results. *We invited 143 bases from 11 countries; 85 (60%) reported base characteristics only and 73 (51%) sample-day data too. The variety of base characteristics was enormous; that is, the target population ranged from 94.000 to 4.500.000. Of 158 requested primary missions, 62 (0.82 per base) resulted in landing. Cardiac aetiology (36%) and trauma (36%) prevailed, mostly of life-threatening severity (43%, 0.64 per mission). Had HEMS been not dispatched, patients would have been attended by another physician in 67% of cases, by paramedics in 24%, and by nurses in 9%. On-board physicians estimated to have caused a major decrease of death risk in 47% of missions, possible decrease in 22%, minor benefit in 17%, no benefit in 11%, and damage in 3%. Earlier treatment and faster transport to hospital were the main reasons for benefit. The most frequent therapeutic procedure was drug administration (78% of missions); endotracheal intubation occurred in 25% of missions and was an option hardly offered by ground crews.* Conclusions. *The study proved feasible, establishing an embryonic network of European HEMS. The participation rate was low and limits the generalizability of the results. Fortunately, because of its cross-sectional characteristics and the handy availability of the web platform, the study is easily repeatable with an enhanced network.

## 1. Introduction

Helicopter emergency medical services (HEMS) are widely diffused in developed countries. However, their actual effectiveness in comparison with ground emergency medical services (GEMS) remains controversial and more research is usually advocated [1].

Research in this field is complicated by the fact that the HEMS and GEMS definitions do not comprise some underlying components with profound influence on patient outcome, like, for example, the type of crew. Therefore, wide-scale comparisons between the two systems have often been impaired by heterogeneity of these important factors. For example, one of the largest studies on the subject grouped together crews led by nurse, paramedic, or physician [2]. To avoid this, it is recommended to document and investigate with as much detail as possible all the elements comprised under the HEMS and GEMS “labels,” ideally as far as the single clinical maneuvers [3–6]. Furthermore, the comparative effectiveness of HEMS versus GEMS is likely to be influenced also by other system-specific, or even mission-specific, characteristics that are difficult to measure and account for in retrospective multicentric analyses (e.g., urban, remote or hostile environment, territorial or demographic distribution of aircrafts/vehicles, etc.).

For these reasons we decided to launch a study aiming at gathering detailed epidemiological data on HEMS missions in Europe. Such information could be important to establish common standards and guidelines in the continent. Moreover, it could promote homogeneity in future comparisons of effectiveness of HEMS versus GEMS and help shed light on their actual determinants (e.g., clinical maneuvers, speed of transport, etc.) netted off against the peculiar characteristics of the single services and missions.

## 2. Material and Methods

This study was conceived and conducted within the European Prehospital Research Alliance (EUPHOREA) network. EUPHOREA is an informal network of European researchers aiming at promoting research in prehospital critical care. More information is available at http://www.euphorea.net.

The design of the study was cross-sectional; it took place in November 15, 2013. The participants were physician-staffed HEMS bases in Europe.

Initially, we developed a questionnaire that consisted of three parts collecting information on (1) the characteristics of the participating HEMS bases, (2) the activities of the study's day, and (3) the characteristics of the missions undertaken during the day. This last part investigated in detail the accomplished missions and their actual effectiveness over GEMS in the local context, as judged by the compiling physician. The questionnaire was meant to be filled by the on-duty HEMS physicians at the end of the working day or soon afterwards, and the approximate time required for compilation was 20 minutes. The questionnaire did not contain personal data of patients. A web site was developed for questionnaire compilation and data recording. The site was password-protected, offering three levels of increasing restrictions for administrator, country coordinator, and base representative. The web system featured also a user's guide and some automatic controls to prevent typos. A mock data collection involving three volunteering HEMS bases took place in August 2013. Based on the experience of this trial and on the comments of the participants, the questionnaire and the web system were finally refined.

Meanwhile, we contacted by email all the HEMS physicians whose addresses were available within the EUPHOREA group and who could be potentially interested. We offered participation in the study and provided the required information. Permissions from the pertinent local authorities (e.g., privacy authorities) were then obtained at discretion of the participants according to the country legislation and habits.

The integral version of the questionnaire is available as additional material.

## 3. Results

We contacted and offered participation to 143 HEMS bases of 11 countries (21 in Austria, 10 in Czech Republic, 2 in Denmark, 5 in Finland, 43 in France, 7 in Hungary, 5 in Italy, 17 in Norway, 26 in Spain, 4 in Sweden, and 3 in the United Kingdom).

The participation rate was different according to the completeness of compilation. Eighty-five bases (60%) filled at least the data of part one, regarding the base characteristics (12 in Austria, 8 in Czech Republic, 2 in Denmark 2, 5 in Finland, 31 in France, 6 in Hungary, 4 in Italy, 4 in Norway, 8 in Spain, 3 in Sweden, and 2 in the United Kingdom). Seventy-three bases (51%) filled also the data on the study's day and missions (11 in Austria, 7 in Czech Republic, 2 in Denmark 2, 2 in Finland, 26 in France, 6 in Hungary, 3 in Italy, 3 in Norway, 8 in Spain, 3 in Sweden, and 2 in the United Kingdom).

The characteristics of the participating bases are shown in Table 1. There was a great variability among the participants, as indicated by the ample ranges, sometimes differing by a factor as large as 100.

In addition to the physician, the medical part of the crew included a nurse in 56 bases (66%), a paramedic in 22 (26%), and a non-health-professional in 7 (8.2). The analysis by country showed that nurses were present in Austria, Czech Republic, France, Hungary, Italy, Norway, Spain, and Sweden. Paramedics were present in Austria, Denmark, Finland, Hungary, Norway, and the United Kingdom. Non-health-professionals were represented in Austria and France.

As for the characteristics of the study's day (part 2 of the questionnaire), the scheduled operating time was (mean ± SD) 13.6 hours, with a min–max range of 6.15–24 hours. The actual operating time due to disruptions of the service for any reason was (mean ± SD) 10.9 hours, with a min–max range of 0–24 hours.  Table 2 displays the workload of the day in terms of number and type of missions. It is striking that, on average, less than one primary mission was completed in one day.

The third part of the questionnaire gathered data on all accomplished missions. The percentages are calculated on the total of bases that filled each data point. This total may be fluctuating because some data fields were left blank.

The most frequent diseases were trauma (36% of missions) and acute cardiac disease (36% of missions), followed by neurological problems (16% of missions).  Table 3 displays the disease severity of the patients. The majority of the patients were found in life-threatening conditions. The definition of life threatening versus non-life-threatening was left to the intervening physician's judgment.

The medical expertise of the counterfactual GEMS facility, that is, the one that in theory would have intervened if the HEMS had not been available for any reason, was (1) physician-level in 51 (67%) missions, (2) paramedic-level in 18 (24%), and (3) nurse-level in 7 (9%). The outcomes of the missions were (1) transport to hospital by HEMS (70%), (2) transport to hospital by HEMS crew on ground ambulance (11%), (3) patient left on scene because of death (11%), and (4) patient left to GEMS crew (8%). The compiling physicians judged that their intervention, as compared to the counterfactual GEMS facility, had caused (1) a major decrease in the risk of death or long-term disability in 47% of missions, (2) a possible minor decrease in the risk of death or long-term disability in 22% of missions, (3) some kind of other benefits to the patients in 17% of missions, (4) no benefit in 11% of missions, and (5) an increase in the risk of death or long-term disability in 3% of missions. The supposed reasons for the improved outcome are shown in Table 4.

The clinical procedures performed by HEMS crews are shown in Table 5.

Among the above therapeutic procedures, the ones that most frequently would have not been performed by the counterfactual GEMS facility were (1) drug-assisted tracheal intubation (in 77% of instances it would have not been performed), (2) central and intraosseous line (50% of instances), (3) drug administration (30% of instances), and (4) peripheral IV line (29% of instances).

The diagnostic procedures performed by HEMS crews are shown in Table 6.

Among the above diagnostic procedures, the ones that most frequently would have not been performed by the counterfactual GEMS facility were (1) invasive monitoring (in 100% of instances it would have not been performed), (2) ultrasound (50% of instances), (3) clinical examination (24% of instances), and (4) peripheral IV line (15% of instances).

## 4. Discussion

We gathered a considerable amount of information from a sample of European HEMSs.

In this pilot edition of the study the participation rate was low (50%) and some important European countries, for example, Germany, were not represented. The validity of the conclusions is therefore inevitably limited. However, the study proved feasible, and because of its cross-sectional design and the web-system already set up, it is easily repeatable in the close future incorporating also some suggestions received by the participants. Furthermore, to our knowledge, it is the first prospective and multinational research on HEMS and therefore can still provide some preliminary indications.

There was an impressive variability regarding the HEMS base characteristics. To some extent, this variability must be a reflection of the different environments where HEMSs operate. For example, HEMSs placed in densely inhabited metropolitan areas will have a large target population and a small operational area. The opposite should occur when HEMS bases serve remote or rural areas. Moreover, as for the number of missions in 2013, some of the answers in the low range could be from new HEMSs opening their activities over the course of the year. However, such an enormous variability should be further investigated in the pursuit of common and agreed standards.

The health crew composition was also variable, with nurses, paramedic, and nonprofessional represented in decreasing order besides the physician. This aspect is also worth further research in order to define the crew composition that gives the best results.

Another striking result was that, on average, less than one mission was accomplished during the study's day. The fact that the study took place in late autumn, when the weather conditions can be unfavorable in Europe, must have played a role. This is also confirmed by an approximate 30% difference between the scheduled and the actual operating times. However, such a small number of missions should also raise some concerns about the actual cost effectiveness of HEMS. In this respect, it will be interesting to repeat the data collection in different seasons.

It is instead comforting that in the relative majority of missions (43%) patients were in life-threatening conditions, indicating a good specificity of the dispatch protocols. Only in 11% of missions were there no medical problems, showing overtriage of dispatch protocols. Furthermore, some of these last missions could have been deliberately dispatched for pure logistic reasons (e.g., mountain rescue). However, in absence of population-based data (i.e., including cases without HEMS involvement), the sensitivity of the protocols cannot be estimated and the full picture remains unknown.

As for the HEMS versus GEMS effectiveness, the participants estimated that their intervention had caused some kind of benefit in the vast majority of cases (nearly 90%). Our data showed also that in Europe physicians are well represented on ground facilities (67% of missions). This could explain why the above-mentioned benefit was attributed predominantly to earlier arrival at scene (52% of cases) and faster transport to hospital (39% of cases), that is, the nonclinical components of HEMS. The items corresponding to better clinical management were chosen less frequently (85%, summing up therapeutic, diagnostic, and triage management). It is worth specifying that the answers to this question were not mutually exclusive, and therefore the percentages do not add up to 100.

The most frequent procedures were drug administration and IV line placement. Tracheal intubation was the fourth most frequent therapeutic procedure (25% of accomplished missions). However, it was also the one that most frequently would have not been performed by GEMS. This indicated that the skills of GEMS physicians might be suboptimal. Other invasive HEMS procedures such as central venous line or chest decompression were performed quite seldom (resp., 4% and 2%). However these percentages should be taken with great caution, given the relatively small number of missions included in the study.

One of the main strengths of this study is, as already said, its multicentric and multinational design. For the first time some common epidemiological data on European HEMS activities were collected. Another strength is that the evaluation of HEMS effectiveness dug deep into the individual HEMS components and was netted off against the local peculiarities. This was made possible by the data collected by mission partakers in nearly real time. This came at the cost of possible bias due to the fact that on-board physicians may have overrated their activities. However, it adds to the compilers' objectivity that in some instances they admitted their intervention caused harm, probably due to complications of invasive clinical maneuvers.

Another strength of the study is the cross-sectional design that facilitated synchronous and homogeneous data collection among many participants from a large territory. However, the cross-sectional design may also have introduced some bias because daily HEMS activities are likely to present cyclic systematic differences (e.g., seasonal or weekly differences in weather, traffic, people activities, etc.). Only when the study will be repeated with pooled data from different periods of the year/week will this bias be eliminated. Another important limitation of the study was the limited number of participants in this pilot edition. However, when repeated with better preparation and, hopefully, increased participation, it should provide further interesting data.

## Supplementary Material

Integral version of the web-based questionnaire.

## Figures and Tables

**Table 1:** Characteristics of HEMS bases.

Base characteristic	Median, interquartile range min–max range
Operational area, km^2^	9000, 6000–15000 700–98900
Target Population, number	675000, 40000–120000 25000–4500000
Number of missions undertaken in 2013	628, 477–839 93–3254

**Table 2:** Frequency and type of missions during the study's day.

Type of missions	Frequency, median (min–max range)
Requested primary missions	158, 2.2 (0–10)
Accepted primary missions	122, 1.5 (0–9)
Aborted primary missions	26, 0.36 (0–4)
Accomplished primary missions	62, 0.82 (0–8)
Secondary interhospital missions	39, 0.54 (0–4)

**Table 3:** Disease severity, by patient and by accomplished mission.

Disease severity	Number of patients (%)	Mean number of patients per mission
No medical problems	10 (11)	0.16
Non-life-threatening disease	34 (36)	0.55
Life-threatening disease	40 (43)	0.64
Cardiac arrest, resuscitation attempted	6 (0.6)	0.09
Cardiac arrest, no resuscitation attempted	3 (0.3)	0.05

**Table 4:** Reasons for theoretical benefits over counterfactual GEMS facility as judged by the helicopter-based physician.

Reason for estimated benefit	Frequency (% of answers) (% of missions^*^)
Earlier arrival at scene and earlier start of procedures that, however, would have been performed also by the alternative ground team, though with some delay	32 (27) (52)
Transportation to the same hospital as ground ambulance, but in less time	24 (20) (39)
Therapeutic interventions not otherwise performed	23 (19) (38)
Diagnostic interventions and early diagnosis not otherwise performed	16 (14) (26)
Transportation to a more appropriate hospital, while the ground facility would have gone to another, less appropriate hospital	13 (11) (21)
Correction of potentially harmful interventions poorly performed by personnel at scene	6 (5) (10)
Pure logistic advantages (e.g., rescue in hostile environment, mountain rescue)	4 (3) (7)

^*^Answers were not mutually exclusive; that is, more than one reason could be given. Only missions where some degree of benefit was estimated to be present were considered for percentages.

**Table 5:** Clinical procedures performed by HEMS crews during the study's day.

Clinical procedure	Frequency (% of answers) (% of missions^*^)
Drug administration	40 (35) (78)
Peripheral IV line	27 (23) (53)
Other: specify^†^	17 (15) (33)
Drug-assisted tracheal intubation	13 (11) (25)
Mechanical external chest compressions	5 (4) (10)
Defibrillation	4 (3) (8)
Supraglottic airway devices or tracheal intubation without drugs	2 (2) (4)
Central IV line	2 (2) (4)
Intraosseous access	2 (2) (4)
Chest decompression (percutaneous, tube, or open)	1 (1) (2)
Cardioversion	1 (1) (2)
Local anesthetic block or infiltration	1 (1) (2)
Surgical airway	0 (0) (0)
Pacing	0 (0) (0)
Pericardial percutaneous drainage	0 (0) (0)
Clamshell thoracotomy	0 (0) (0)

IV: intravenous.

^*^Answers were not mutually exclusive; that is, more than one reason could be given. Denominators of percentages are only cases for which at least one answer was ticked.

^†^Examples include urinary catheterization, limb immobilization, aerosol, and pelvic belt.

**Table 6:** Diagnostic procedure performed by HEMS during the study's day.

Diagnostic procedure	Frequency (% of answers) (% of missions^*^)
Clinical examination	37 (43) (79)
ECG analysis (12 leads)	27 (31) (57)
Point-of-care lab tests	8 (9) (17)
Invasive monitoring	6 (7) (13)
US/Doppler	5 (6) (11)
Other: specify^†^	4 (5) (9)

ECG: electrocardiography; US: ultrasound.

^*^Denominators of percentage are only cases for which at least one answer was ticked.

^†^Examples include noninvasive monitoring (e.g., blood pressure).
