# Supplementary material for: Cross-Sectional Investigation of HEMS Activities in Europe: A Feasibility Study
Source: ScientificWorldJournal. 2014 Nov 30;2014:201570. doi: 10.1155/2014/201570 (PMC4265707; doi:10.1155/2014/201570)
Supplement: Supplementary file 1 — Integral version of the web-based questionnaire. [file 201570.f1.docx]

Questionnaire

Part 1. Main features of participating HEMS base

1. Name of HEMS base:

2. Country: Austria, Czech Republic, Denmark, Finland, Germany, England, Hungary, Italy, Norway, Scotland, Sweden, Switzerland

3. Operational area (approximate, Km²)

4. Population covered by HEMS base (approximate number of persons)

5. Approximate number of missions undertaken in year 2012

6. Medical expertise of crewmembers:

1) Physician + nurse

2) Physician + paramedic

3) Physician + non health-professional (i.e. aeronautic or mountain rescue personnel)

4) Physician not present onboard

7a. Name and Surname of compiling physician

7b. Email

7c. Telephone number

8 Notes to part 1.

Part 2. Characteristics of the sample day

1. Scheduled operating time in the sample day (hh.mm.)

*Planned availability of HEMS service during the sample day*

2. Actual operating time in the sample day (hh.mm.)

*Actual availability of HEMS service during the sample day in case of any service disruption (e.g. adverse weather conditions, mechanical problems of the aircraft, unexpected problems in general).*

3. Number of requested missions:

4. Number of accepted requests (started missions):

*A request is considered accepted if the helicopter takes off, even if take-off is not from usual HEMS base or the mission is aborted during the flight. Missions assigned during flight should also be counted here and in the previous question*

5. Number of accepted missions aborted before landing for any reason

*A mission is considered aborted when no more necessary (notification by radio) or deemed not feasible by HEMS crew (e.g. bad weather).*

6. Number of accomplished primary missions:

*A primary mission is considered accomplished if the crew land on the ground with the purpose of providing assistance to a patient (excluding interhospital transports, counted in the next question). In rare instances, a mission may be considered accomplished also without landing (e.g. search and rescue with hoisting or without finding the victim)*

7. Number of missions for interhospital transports:

8. Approximate amount of time spent on missions (hh.mm)

*It should correspond to the actual operating time of the sample day (question 8) minus the idle time spent at HEMS base*

9. Approximate amount of time spent on primary missions (hh.mm)

10. Approximate amount of time spent on interhospital missions (hh.mm)

11. Notes to part 2.

Part 3. Mission data (one set for each flight, primary missions only)

1. Level of medical expertise of the ground ambulance or other rescue team present at scene:

1) Physician

2) Nurse

3) Paramedic

4) Non health care professionals (e.g. mountain rescue, police etc.)

5) No one of the above at scene

*Presence at scene is regardless of whether arriving before or later than HEMS. Ignore any bystander with professional skills accidentally happening to be on the scene.*

2. Ground ambulance or rescue team that would have intervened if HEMS had not been dispatched for any reason: level of medical expertise:

1) Physician

2) Nurse

3) Paramedic

4) Non health care professionals

3. Number of patients managed by HEMS crew:

1) Number of patients with no medical problems

2) Number of patients In non life threatening conditions

3) Number of patients In life threatening conditions

4) Number of patients found in cardiac arrest, resuscitation attempted

5) Number of patients found dead, no resuscitation attempted

4. Type of disease detected at scene upon arrival ,

1) Trauma

2) Neurological (consciousness disturbances, seizures, TIA/stroke, etc.)

3) Cardiological (acute coronary syndrome, acute heart failure, rhythm disturbances, etc.

4) Respiratory (COPD exacerbation, asthma, pneumonia, etc.)

5) Abdominal (acute abdominal pain, rupture of aortic aneurism, etc.)

6) Gyn./Obstetrics (bleeding, delivery etc.)

7) Metabolic (hypo/hyper glycemia, thyrotoxic crisis, exhaustion after intense physical activity, etc.)

5. Mission outcome (s)

1) Patient transported to hospital by HEMS

2) Patient transported to hospital by ground ambulance, HEMS physician assisting onboard

3) Patient left to a ground ambulance for transportation to hospital, HEMS physician not assisting onboard

4) Victim in hostile or difficult terrain transported to a safer place, no need

for medical assistance

5) Victim left at scene in good health

6) Patient left at scene because of death

7) No victim found

6. Estimation of the benefit for the patient(s) related to HEMS intervention, compared to the use of ambulances or other ground emergency services. ;

1) Major or significant decrease in death risk or long-term disability

2) Possible minor decrease in death-risk or long-term disability

3) No decrease in death risk or disability but some kind of benefit to the patient

4) No benefit for the patient related to HEMS intervention

5) Increase of death risk or disability

*Answer 3: e.g. better analgesia, rescue from hostile environment. Answer 5: e.g. attempted procedures proving harmful or delaying appropriate treatment*

7. Reasons for patient improved outcome related to HEMS deployment as compared to ground medical response:

1) Therapeutic interventions not otherwise performed

2) Diagnostic interventions and early diagnosis not otherwise performed

3) Earlier arrival at scene and earlier start of procedures that, however, would have been performed also by the alternative ground team, though with some delay

4) Correction of potentially harmful interventions poorly performed by personnel at scene

5) Transportation to a more appropriate hospital, while the ground facility would have gone to another, less appropriate hospital.

6) Transportation to the same hospital as ground ambulance, but in less time

7) Pure logistic advantages (e.g. rescue in hostile environment, mountain rescue)

*Multiple choices allowed*

8. Therapeutic interventions performed on this patient by HEMS team

1) Drug-assisted tracheal intubation

2) Supraglottic Airway Devices or tracheal intubation without drugs

3) Surgical airway

4) Chest decompression (Percutaneous, tube or open)

5) Peripheral IV line

6) Central IV line

7) IO Access

8) Defibrillation

9) Cardioversion

10) Pacing

12) Mechanical external chest compressions

13) Pericardial percutaneous drainage

14) Clamshell thoracotomy

15) Local anesthetic block or infiltration

16) Drug administration

17) Other: specify in free text

*Multiple choices allowed.*

9. Of the above, please indicate which ones would have not been performed by the alternative ground facilities.

1) Drug-assisted tracheal intubation

2) Supraglottic Airway Devices or tracheal intubation without drugs

3) Surgical airway

4) Chest decompression (Percutaneous, tube or open)

5) Peripheral IV line

6) Central IV line

7) IO Access

8) Defibrillation

9) Cardioversion

10) Pacing

12) Mechanical external chest compressions

13) Pericardial percutaneous drainage

14) Clamshell thoracotomy

15) Local anesthetic block or infiltration

16) Drug administration

17) Other: specify in free text

*Multiple choices allowed.*

10. Diagnostic interventions performed on this patient by HEMS team

1) Diagnosis based on clinical examination

2) US/Doppler

3) ECG analysis (12-lead)

4) Invasive monitoring

5) Point-of-care lab tests

6) Other: specify in free text

*Multiple choices allowed.*

11. Of the above, please indicate which ones would have not been performed by the alternative ground facilities.

1) Diagnosis based on clinical examination

2) US/Doppler

3) ECG analysis (12-lead)

4) Invasive monitoring

5) Point-of-care lab tests

6) Other: specify in free text

*Multiple choices allowed.*

11. Notes to part 3.
